# The exploration of optimal gestational weight gain after oral glucose tolerance test for Chinese women with gestational diabetes mellitus

**DOI:** 10.1038/s41598-024-51879-x

**Published:** 2024-01-17

**Authors:** QingXiang Zheng, Yu Zhu, XiuMin Jiang, Ling Huang, JiaNing Li, RuLin Liu

**Affiliations:** 1 Fujian Maternity and Child Health Hospital College of Clinical Medicine for Obstetrics and Gynecology and Pediatrics, Fujian Medical University, Fuzhou, Fujian China; 2 Fujian Obstetrics and Gynecology Hospital College of Clinical Medicine for Obstetrics and Gynecology and Pediatrics, Fujian Medical University, Fuzhou, Fujian China; 3 The School of Nursing, Fujian Medical University, Fuzhou, Fujian China; 4 The School of Nursing, Fujian University of Traditional Chinese Medicine, Fuzhou, Fujian China

**Keywords:** Endocrinology, Health care

## Abstract

Now, no recommendations of gestational weight gain (GWG) after gestational diabetes mellitus (GDM) diagnosis for Chinese women was made. This study aimed to explore the optimal GWG after oral glucose tolerance test (OGTT) for Chinese women with GDM. The GWG status of 11,570 women was retrospectively analyzed. Binary regression model and restricted cubic spline were used to estimate the association between GWG after OGTT and the predicted probability of adverse outcomes. Based on above, the optimal GWG was defined as the range that not exceed 1% increase in the predicted probability from the lowest point. Results shown that every increased one unit GWG after OGTT was associated with higher risks of macrosomia, cesarean section and LGA, and lower risk of preterm birth. According to the WHO and Working Group on Obesity in China (WGOC) recommended pre-pregnancy BMI category, the optimal GWG were proposed: 3.66 to 6.66 kg/3.66 to 6.66 kg in underweight group, 3.07 to 6.50 kg/3.02 to 6.40 kg in normal weight group, 1.06 to 2.73 kg/0 to 1.99 kg in overweight group, and not applicable/− 0.22 to 2.53 kg in obese group, respectively. Therefore, it is necessary to classified Chinese population based on the WGOC recommended pre-pregnancy BMI category, that influenced the contribution of pre-pregnancy BMI groups and the optimal GWG recommendation for GDM women with overweight or obesity.

## Introduction

Gestational diabetes mellitus (GDM), a serious obstetrical complication, is defined as carbohydrate intolerance develops during pregnancy^1^. It affects 13.97–14.04% of pregnancies worldwide^2^, and even reach 14.8% in China^3^. Importantly, GDM will remain be a huge challenge in next decades^4^. Compared with women with normoglycemia, women with GDM were at an increased odds of pregnancy adverse outcomes, such as caesarean section, preterm birth, macrosomia and neonatal hypoglycemia^5,6^. Moreover, women who suffered from GDM were easier to develop type 2 diabetes after delivery^7^ and their offspring also had a significantly increased risk of diabetes^8^. And there were significant economic burdens in China that had been imposed by GDM complications, which reached 19.36 billion per year^9^. Therefore, it indicated an imperative need to pay more attention to prevent and intervene GDM.

Gestational weight gain (GWG) represents an indicator that is to reflect weight change and fetus nutrition status during pregnancy^10^. To promote better perinatal outcomes, the United States National Academy of Medicine (NAM) released recommended guidelines for GWG based on different pre-pregnancy body mass index (BMI) category^11^. According to the NAM recommendations, inappropriate GWG were associated with higher risks of pregnancy adverse outcomes, including macrosomia, large for gestational age (LGA), low birth weight, small for gestational age (SGA) and preterm birth^12^. In 2021, Chinese Nutrition Society (CNS) developed the *Weight Monitoring and Evaluation During Pregnancy Period of Chinese Women* attributed to the limitation of NAM guidelines’ generalizability to Chinese populations^13^. Comparison of NAM and CNS guidelines showed that higher GWG was recommended by the former, whereas the latter was fitter for Chinese women^14^. Plus, evidence have proved the importance of weight management among women with GDM^15^. As such, it is necessary to specialize GWG strategy for Chinese women, especially for GDM women.

However, there were no specific GWG consensus for GDM women now. Our early study^16^ showed that women with GDM in different pre-pregnancy BMI groups have distinct GWG ranges and GWG rates compared with those from the NAM recommendations. Besides, GDM women with excessive GWG were related to higher risks of maternal-infant adverse outcomes than the normal ones^16^. In addition, a systematic review also indicated that GWG above the NAM recommendations among GDM women were associated with higher risks of caesarean section, hypertensive disorders, LGA and macrosomia, while GWG below the NAM recommendations had protective effects on LGA and macrosomia^17^. Bogdanet et al.^18^ suggested that GWG ranges recommended by the NAM were too much for GDM women. Confirmed it, another original study^19^ that explored the GDM-specific optimal GWG of Chinese women illustrated that the optimal GWG for women with normal weight and obesity were both lower than NAM recommendations. Collectively, GWG guidelines for Chinese women with GDM should be proposed specially and separately based on different pre-pregnancy BMI category.

In China, as practice in many other countries, pregnant women were screened GDM via an oral glucose tolerance test (OGTT) during 24th–28th gestational weeks^20^. Women controlled their GWG reference to general GWG guidelines before 24th gestational week, as they were not diagnosed as GDM at that moment. It has been found that GWG before OGTT among Chinese women with GDM has no association with any pregnancy adverse outcomes, however, GWG after GDM diagnosis was positively related to risks of LGA, macrosomia and cesarean section^21^. That signified that GWG after OGTT was more important than GWG before OGTT^21^. A paucity of GWG strategy after GDM diagnosis appeals new explorations. Therefore, this study aimed to derive the optimal GWG after OGTT among Chinese women with GDM.

## Results

**Figure 1:**
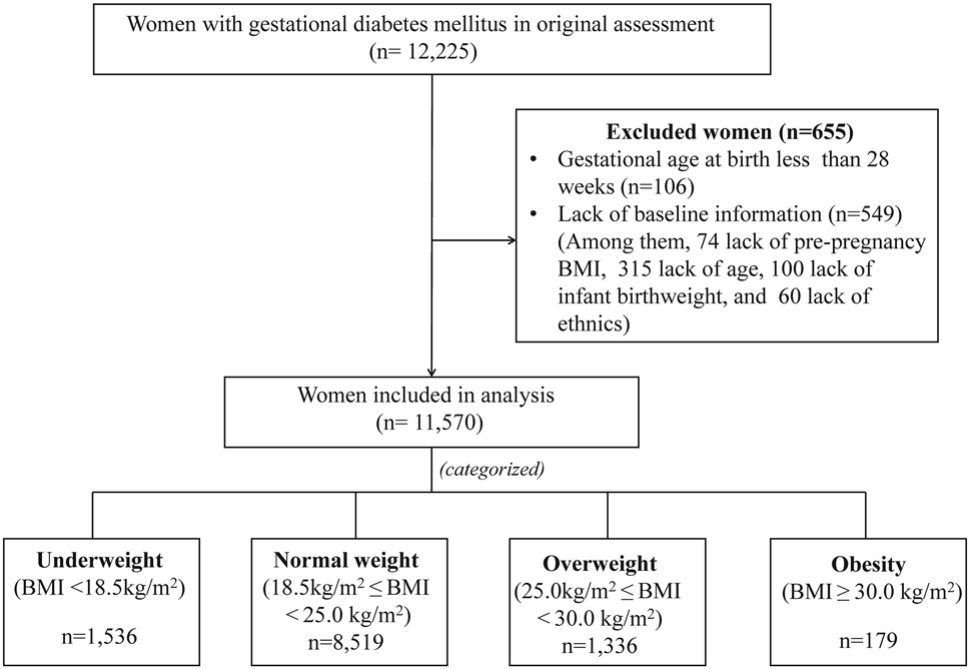
Derivation of the study population.

The participants selection process was presented in Fig. 1 Of 12,225 GDM women, 655 women were excluded due to not accord with the inclusion criteria. Finally, 11,570 participants were included in this study. Table 1 showed the demographic characteristics and pregnancy adverse outcomes of GDM women, and the comparisons of four pre-pregnancy BMI stratifications. There were 1536 (13.28%) women was categorized as in underweight group; 8519 (73.63%) in normal weight group, 1336 (11.55%) in overweight group, and 179 (1.55%) in obesity group. GDM women in underweight group were younger than other groups. Lower GWG (whether before or after OGTT) were associated with higher pre-pregnancy BMI. The median GWG after OGTT and its quartile 1 and 3 were 5.84 kg (4.20 kg and 7.60 kg), 5.00 kg (3.40 kg and 6.87 kg), 4.22 kg (2.50 kg and 6.00 kg), 4.20 kg (2.10 kg and 5.85 kg) in underweight, normal weight, overweight and obesity group, respectively.

**Table 1:** Characteristics of study participants categorized by WHO BMI criteria.

Variables	All participants (n = 11,570)	Underweight group (n = 1,536, BMI < 18.5 kg/m^2^)	Normal weight group (n = 8519, 18.5 kg/m^2^ ≤ BMI < 25.0 kg/m^2^)	Overweight group (n = 1336, 25.0 kg/m^2^ ≤ BMI < 30.0 kg/m^2^)	Overweight group (n = 179, BMI ≥ 30.0 kg/m^2^)	*P* values
Age (year)	31.00 (28.00, 34.00)	28.00 (26.00, 32.00)	31.00 (28.00, 34.00)	32.00 (29.00, 36.00)	31.00 (27.00, 34.00)	< 0.001
Ethnics [N (%)]						
Han nationality	11,441 (98.89)	1522 (99.09)	8418 (98.81)	1324 (99.10)	177 (98.88)	0.706
Minority nationality	129 (1.11)	14 (0.91)	101 (1.19)	12 (0.90)	2 (1.12)	
Pre-pregnancy BMI (kg/m^2^)	21.29 (19.53, 23.25)	17.72 (17.09, 18.02)	21.30 (20.03, 22.78)	26.37 (25.59, 27.50)	31.25 (30.49, 42.89)	< 0.001
Maternal bodyweight at diagnosis of GDM (kg)	62.00 (56.88, 67.90)	53.00 (50.05, 55.93)	62.00 (57.93, 66.45)	73.58 (69.50, 78.33)	85.50 (80.14, 92.09)	< 0.001
Total GWG during pregnancy	12.60 (9.90, 15.50)	13.75 (11.50, 16.20)	12.70 (10.00–15.60)	10.10 (7.08, 13.50)	8.30 (4.85, 12.00)	< 0.001
Maternal GWG before OGTT (kg)	7.50 (5.45, 9.63)	7.90 (6.20, 9.95)	7.65 (5.67, 9.80)	6.00 (3.75, 8.25)	4.25 (1.69, 7.28)	< 0.001
Maternal GWG after OGTT (kg)	5.00 (3.35, 6.90)	5.84 (4.20, 7.60)	5.00 (3.40, 6.87)	4.22 (2.50, 6.00)	4.20 (2.10, 5.85)	< 0.001
Gestational age at birth (week)	39.00 (38.00, 40.00)	39.00 (38.00, 40.00)	39.00 (38.00, 40.00)	39.00 (38.00, 40.00)	39.00 (38.00, 40.00)	0.287
Infant birthweight (kg)	3295.00 (3000.00, 3580.00)	3180.00 (2910.00, 3440.00)	3300.00 (3005.00, 3580.00)	3370.00 (3054.00, 3700.00)	3480.00 (3155.00, 3775.00)	< 0.001
Outcomes [N (%)]						
Macrosomia	638 (5.51)	32 (2.08)	450 (5.28)	132 (9.88)	24 (13.41)	< 0.001
Preterm	1022 (8.83)	113 (7.36)	771 (9.05)	123 (9.21)	15 (8.38)	0.177
Cesarean section	4169 (36.03)	356 (23.18)	3089 (36.26)	626 (46.86)	98 (54.75)	< 0.001
Gestational hypertension	187 (1.62)	10 (0.65)	124 (1.46)	41 (3.07)	12 (6.70)	< 0.001
LGA	1162 (10.04)	69 (4.49)	841 (9.87)	215 (16.09)	37 (20.67)	< 0.001
SGA	899 (7.77)	187 (12.17)	621 (7.29)	79 (5.91)	12 (6.70)	< 0.001

*GWG* gestational weight gain, *OGTT* oral glucose tolerance test, *LGA* large for gestational age, *SGA* small for gestational age.

**Figure 2:**
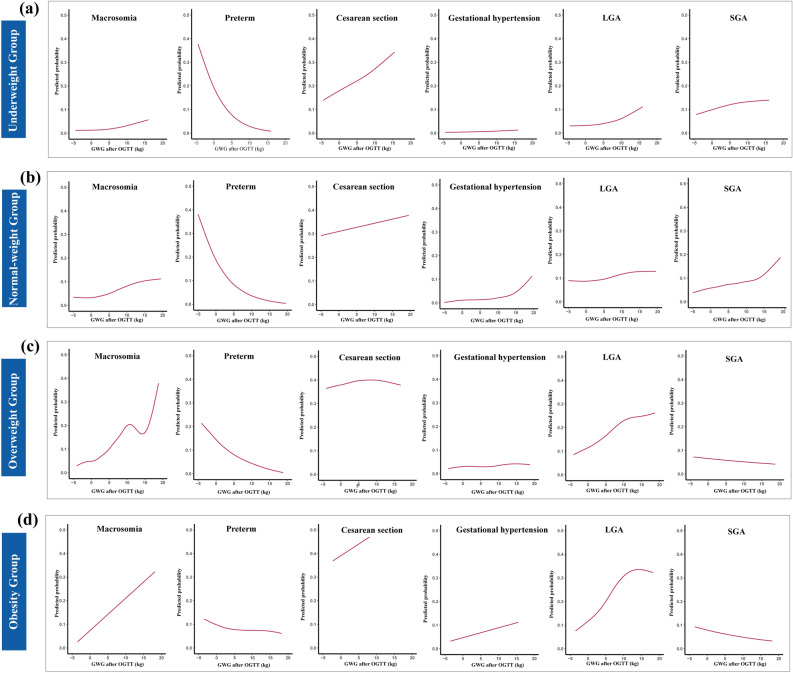
Association between gestational weight gain after oral glucose tolerance test and the predicted probability of adverse outcomes in different pre-pregnancy BMI categories. Binary regression model with lowest Akaike information criterion value was used to calculate predicted probability of adverse outcomes including macrosomia, preterm birth, cesarean section, gestational hypertension, LGA and SGA. *GWG* gestational weight gain, *OGTT* oral glucose tolerance test, *LGA* large for gestational age, *SGA* small for gestational age. (**a**) Underweight group; (**b**) Normal weight group; (**c**) Overweight group; (**d**) Obesity group.

In general, the crude model performed better in the underweight group with lower AIC values, whereas logit link model performed better in normal weight and overweight group. However, no model showed better performance in obesity group. The predicted probability of each adverse outcome was proposed based on the “best-fit” model with lowest AIC value among three models. The fit curve that showing the association of GWG after OGTT and the predicted probability of pregnancy adverse outcomes were illustrated in Fig. 2. In majority of groups, the predicted probability of macrosomia, LGA, cesarean section and gestational hypertension were increased with higher GWG after OGTT, however, preterm birth probability was decreased with higher GWG after OGTT. Besides, positive relationship between GWG after OGTT and SGA probability was showed in underweight and normal weight group, whereas negative correlation between them was found in overweight and obesity group.

As shown in Table 2, across all pre-pregnancy BMI categories, higher GWG after OGTT was related to higher risk of LGA and lower risk of preterm birth (*P* < 0.05), however, no significant association was found between GWG after OGTT and SGA (*P* > 0.05). Except for GDM women in underweight group, higher GWG after OGTT was associated with higher risk of macrosomia (*P* < 0.05). Beside to GDM women in obesity group, GWG after OGTT was positively associated with higher risk of cesarean section (*P* < 0.05). The relationship between higher GWG after OGTT and increased risk of gestational hypertension was only found in the normal weight group (*P* < 0.05).

**Table 2:** The predictive probability of macrosomia, preterm, cesarean section, gestational hypertension, LGA and SGA in association with maternal gestational weight gain after oral glucose tolerance test for underweight, normal weight and overweight GDM women by different model.

	Crude			Logit			Complementary log–log		
	OR (95% CI)	*P*	AIC	Adjusted OR (95% CI)	*P*	AIC	Adjusted OR (95% CI)	*P*	AIC
Underweight									
Macrosomia	1.11 (0.98–1.25)	0.092	312.37	1.11 (0.98–1.25)	0.013	314.29	1.11 (0.98–1.24)	0.102	314.28
Preterm birth	0.24 (0.16–0.35)	< 0.001	773.18	0.81 (0.75–0.87)	< 0.001	776.86	0.84 (0.79–0.89)	< 0.001	778.84
Cesarean section	1.06 (1.02–1.11)	0.008	1660.10	1.11 (1.06–1.16)	< 0.001	1626.40	1.09 (1.05–1.13)	< 0.001	1626.10
Gestational hypertension	1.07 (0.86–1.32)	0.535	124.24	1.13 (0.91–1.36)	0.226	125.50	1.13 (0.91–1.33)	0.226	125.10
LGA	1.08 (0.99–1.17)	0.095	564.28	1.10 (1.01–1.19)	0.032	554.08	1.09 (1.01–1.18)	0.034	554.40
SGA	1.03 (0.98–1.09)	0.277	1140.60	1.02 (0.96–1.08)	0.612	1117.10	1.01 (0.96–1.07)	0.640	1116.50
Normal weight									
Macrosomia	1.10 (1.07–1.14)	< 0.001	3494.30	1.10 (1.06–1.14)	< 0.001	3289.80	1.10 (1.06–1.13)	< 0.001	3290.30
Preterm birth	0.83 (0.81–0.85)	< 0.001	5000.90	0.84 (0.81–0.86)	< 0.001	4977.80	0.86 (0.84–0.88)	< 0.001	4989.40
Cesarean section	1.02 (1.01–1.04)	0.002	11,152.00	1.09 (1.09–1.23)	< 0.001	10,623.00	1.07 (1.05–1.07)	< 0.001	10,629.00
Gestational hypertension	1.08 (1.02–1.15)	0.013	1293.20	1.18 (1.10–1.26)	< 0.001	1246.20	1.16 (1.09–1.13)	< 0.001	1246.30
LGA	1.03 (1.01–1.06)	0.010	5488.10	1.06 (1.03–1.09)	< 0.001	5247.00	1.06 (1.03–1.08)	< 0.001	5249.20
SGA	1.04 (1.01–1.07)	0.005	4444.10	1.03 (1.00–1.06)	0.060	4376.90	1.03 (1.00–1.06)	0.067	4377.00
Overweight									
Macrosomia	1.16 (1.09–1.23)	< 0.001	841.7	1.14 (1.07–1.21)	< 0.001	806.03	1.12 (1.06–1.19)	< 0.001	806.36
Preterm birth	0.88 (0.82–0.93)	< 0.001	807.35	0.88 (0.83–0.94)	< 0.001	806.87	0.91 (0.87–0.95)	< 0.001	808.56
Cesarean section	1.03 (0.99–1.06)	0.173	1848.90	1.04 (1.01–1.08)	0.026	1783.30	1.03 (1.01–1.06)	< 0.018	1783.50
Gestational hypertension	1.01 (0.91–1.12)	0.833	370.36	1.05 (0.94–1.17)	0.385	360.74	1.05 (0.94–1.16)	0.384	360.92
LGA	1.08 (1.03–1.14)	0.001	1172.50	1.09 (1.04–1.15)	< 0.001	1146.70	1.08 (1.03–1.13)	< 0.001	1147.20
SGA	0.97 (0.91–1.05)	0.500	603.61	0.97 (0.90–1.04)	0.373	605.94	0.97 (0.90–1.04)	0.371	605.92
Obesity									
Macrosomia	1.14 (1.01–1.28)	0.033	140.27	1.11 (0.99–1.26)	0.096	141.00	1.10 (0.99–1.22)	0.061	140.58
Preterm birth	0.97 (0.88–1.09)	0.521	106.72	0.97 (0.88–1.09)	0.550	111.55	0.97 (0.90–1.09)	0.565	111.48
Cesarean section	1.06 (0.99–1.15)	0.148	248.18	1.06 (0.98–1.40)	0.130	247.48	1.05 (0.99–1.11)	0.110	246.75
Gestational hypertension	1.12 (0.96–1.30)	0.153	90.00	1.16 (0.98–1.40)	0.098	90.05	1.12 (0.96–1.25)	0.146	90.60
LGA	1.16 (1.05–1.30)	0.006	178.00	1.15 (1.03–1.30)	0.016	172.88	1.11 (1.02–1.20)	0.017	173.55
SGA	0.95 (0.86–1.07)	0.328	91.22	0.95 (0.85–1.07)	0.301	87.638	0.96 (0.90–1.06)	0.322	88.07

Data are represented as OR (95% CI). ORs were calculated by binary regression model using crude, “logit” and “complementary log–log” links, and were adjusted for age, pre-pregnancy BMI, gestational age at birth, GWG before OGTT. Particularly, gestational age at birth was not adjusted in LGA, SGA and preterm birth models.

*OR* odds ratio, *CI* confidence interval, *AIC* Akaike information criterion, *GWG* gestational weight gain, *OGTT* oral glucose tolerance test, *LGA* large for gestational age, *SGA* small for gestational age. Underweight, normal weight, overweight and obesity were defined as: pre-pregnancy BMI < 18.5 kg/m^2^, 18.5 kg/m^2^ ≤ BMI < 25.0 kg/m^2^, 25.0 kg/m^2^ ≤ BMI < 30.0 kg/m^2^ and BMI ≥ 30.0 kg/m^2^, respectively.

**Figure 3:**
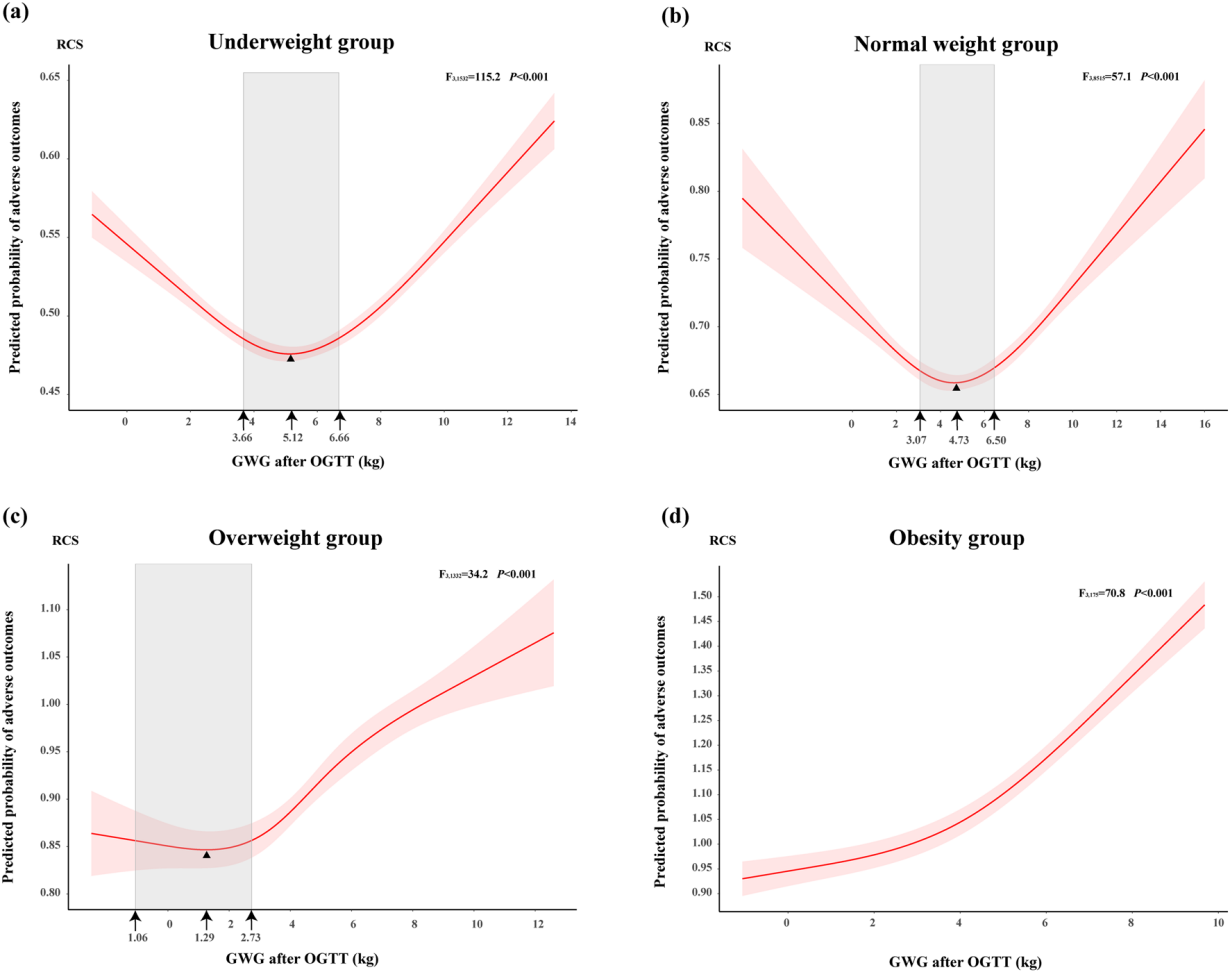
Predicted probability of adverse pregnancy outcomes by gestational weight gain after oral glucose tolerance test (Pre-pregnancy BMI category based on WHO criteria). The red solid curve represents predicted probability of adverse outcomes of macrosomia, preterm birth, cesarean section, gestational hypertension, LGA and SGA, and red shade represents 95% confidence interval of predicted probability of adverse outcomes. The grey shad indicates optimal GWG after OGTT in every pre-pregnancy BMI category group. Triangle represents the lowest predictive probability of adverse outcomes. Values that arrow points to represents the lower limit of optimal GWG, GWG with the lowest probability of adverse outcomes and the upper limit of optimal GWG, from left to right. GWG, gestational weight gain; OGTT, oral glucose tolerance test. (**a**) Underweight group; (**b**) Normal weight group; (**c**) Overweight group; (**d**) Obesity group.

Figure 3 showed the nonlinear relationships between the predicted probability of adverse outcomes and GWG after OGTT in four pre-pregnancy BMI categories, which were analyzed by RCS. In underweight and normal weight group, the predicted probability of adverse outcomes showed a U-shape curve with increased GWG after OGTT (*F*_3,1532_ = 115.2, *P* < 0.001; *F*_3,8515_ = 57.1, *P* < 0.001, respectively). In overweight group, the predicted probability of adverse outcomes decreased gradually first and then increased rapidly (*F*_3,1332_ = 34.2, *P* < 0.001). However, in obesity group, predicted probability of adverse outcomes increased with higher GWG after OGTT (*F*_3,175_ = 70.8, *P* < 0.001). For four pre-pregnancy BMI categories, the predicted probability of adverse outcomes was a comprehensive result gathering six pregnancy adverse outcomes. Figure 3 also depicted the optimal GWG after OGTT, which was defined as the range bounded the 1% rise in the lowest adverse outcomes probability^22^ and was represented as shaded area. For instance, the predicted probability of adverse outcomes among GDM women in underweight group was lowest when GWG after OGTT was 5.12 kg, and the optimal GWG after OGTT was 3.66–6.66 kg. For GDM women in normal weight group, the lowest adverse outcomes probability appeared at GWG after OGTT with 4.73 kg, and the optimal range was 3.07–6.50 kg. When comes to the overweight group, GDM women with 1.29 kg of GWG after OGTT had lowest adverse outcomes probability, and its optimal range was 1.06–2.73 kg. However, we could not propose the optimal GWG for GDM women with obesity due to the predicted probability of adverse outcomes increased with a single trend.

**Figure 4:**
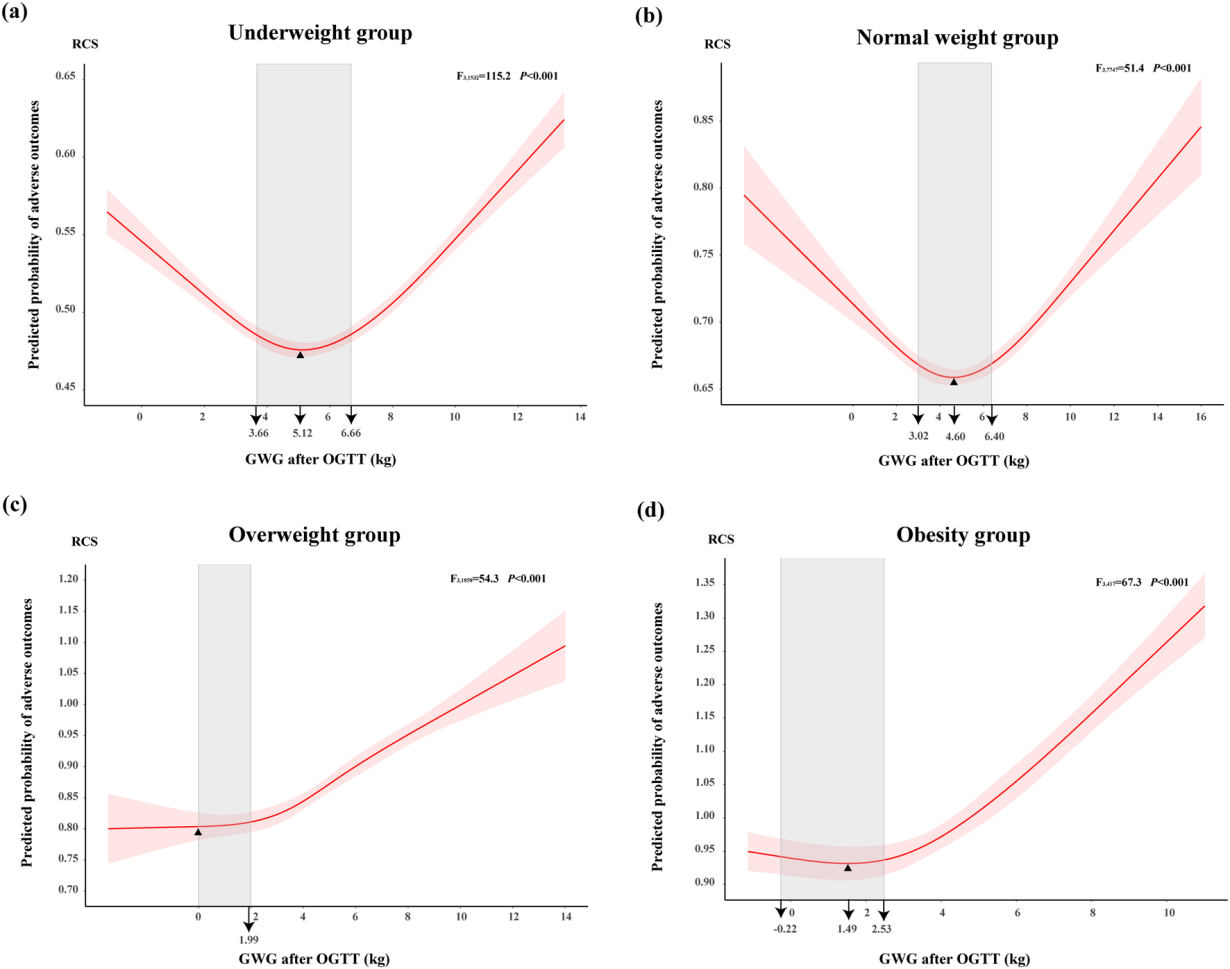
Predicted probability of adverse pregnancy outcomes by gestational weight gain after oral glucose tolerance test (Pre-pregnancy BMI category based on WGOC criteria). The red solid curve represents predicted probability of adverse outcomes of macrosomia, preterm birth, cesarean section, gestational hypertension, LGA and SGA, and red shade represents 95% confidence interval of predicted probability of adverse outcomes. The grey shad indicates optimal GWG after OGTT in every pre-pregnancy BMI category group. Triangle represents the lowest predictive probability of adverse outcomes. Values that arrow points to represents the lower limit of optimal GWG, GWG with the lowest probability of adverse outcomes and the upper limit of optimal GWG, from left to right. *GWG* gestational weight gain, *OGTT* oral glucose tolerance test, *WGOC* the Working Group on Obesity in China. (**a**) Underweight group; (**b**) Normal weight group; (**c**) Overweight group; (**d**) Obesity group.

Pre-pregnancy BMI in this study was calculated according to the WHO recommendations^23^. However, given to the Working Group on Obesity in China (WGOC) has developed tailored BMI classification criteria for Chinese^24^: underweight (BMI < 18.5 kg/m^2^), normal weight (18.5 kg/m^2^ ≤ BMI < 24.0 kg/m^2^), overweight (24.0 kg/m^2^ ≤ BMI < 28.0 kg/m^2^), and obesity (BMI ≥ 28.0 kg/m^2^), we also performed another exploration of the optimal GWG after OGTT based on WGOC criteria. Using pre-pregnancy BMI classification of WGOC, less women were sorted to normal weight group and more women were sorted to overweight and obese group. Overall, the results of performance based on WGOC criteria remained stable and detailed data were reported in Supplementary Tables S1, S2 and Figs. S1, S2. We developed the optimal GWG range as follows: (3.66 to 6.66) kg for underweight group, (3.02 to 6.40) kg for normal weight group, (0 to 1.99) kg for overweight group and (− 0.22 to 2.53) kg for obese group (Table 3 and Fig. 4). Notably, in overweight group, we set lower limit of the optimal GWG after OGTT as 0 kg due to the predicted probability remained lowest steadily when GWG after OGTT below 0 kg, with 0.80 (Fig. 4). Compared with the optimal GWG after OGTT developed based on WHO criteria, WGOC criteria customized lower GWG recommendation for women with overweight and limited GWG recommendation range for women with obesity, respectively. Besides, the optimal GWG after OGTT that proposed based on WGOC criteria recommend women with overweight and obesity to loss their weight after GDM diagnosis.

**Table 3:** Optimal gestational weight gain after oral glucose tolerance test with lowest probability of adverse outcomes, stratified by pre-pregnancy BMI according to WHO criteria and WGOC criteria, and GWG reference for Chinese women from earlier study.

Pre-pregnancy BMI category	Optimal GWG after OGTT for Chinese women with GDM in this study (kg)		Optimal GWG recommendations from earlier studies for Chinese women (kg)		
	WHO criteria	WGOC criteria	Cheng et al.^40^	Zhang et al.^22^	He et al.^48^
Underweight group	3.66 to 6.66	3.66 to 6.66	NA	15.0 to 19.5	12·8 to 17·1
Normal weight group	3.07 to 6.50	3.02 to 6.40	9.1 to 14.3	12.0 to 18.5	12·1 to 16·4
Overweight group	1.06 to 2.73	0 to 1.99	2.7 to 7.6	6.5 to 12.0	10·4 to 14·9
Obesity group	NA	− 0.22 to 2.73	NA	NA	NA

WHO criteria: underweight (BMI < 18.5 kg/m^2^), normal weight (18.5 kg/m^2^ ≤ BMI < 25.0 kg/m^2^), overweight (25.0 kg/m^2^ ≤ BMI < 30.0 kg/m^2^), and obesity (BMI ≥ 30.0 kg/m^2^).

WGOC criteria: underweight (BMI < 18.5 kg/m^2^), normal weight (18.5 kg/m^2^ ≤ BMI < 24.0 kg/m^2^), overweight (24.0 kg/m^2^ ≤ BMI < 28.0 kg/m^2^), and obesity (BMI ≥ 28.0 kg/m^2^).

Cheng et al. explore the optimal total GWG during pregnancy for Chinese women with GDM based on WGOC pre-pregnancy BMI criteria. Zhang et al. and He et al. explored the optimal total GWG during pregnancy for Chinese women based on WGOC pre-pregnancy BMI criteria.

*GDM* gestational diabetes mellitus, *GWG* gestational weight gain, *OGTT* oral glucose tolerance test, *WGOC* Working Group on Obesity in China, *NA* not available.

## Discussion

This study found that total GWG after OGTT decreased with the increasing pre-pregnancy BMI in four pre-pregnancy BMI categories, which was consistent with the results of previous studies conducted by Zhang et al.^22^ among normal women, and Miao et al.^25^ among GDM women. Such distribution was also reflected in GWG before OGTT in this present study, as well as in accordance with Hong et al.’s results among GDM women. Besides, prior investigation suggested that women with GDM has less total GWG compared with normal women due to less GWG rates after GDM screening^26,27^. This phenomenon might result from that GDM women received lifestyle modification such as diet control and weight management following GDM diagnosis. Furthermore, it also indicated that GWG after GDM diagnosis need to be is considered additionally.

Evidence have proved the importance of weight management during pregnancy among women with GDM^15,28,29^. Aiken et al.^30^ revealed that controlling GWG after GDM diagnosis made more sense for GDM women since GWG before GDM diagnosis was not related to any pregnancy adverse outcomes, while higher GWG after OGTT was associated with higher risks of LGA and instrumental delivery. Furthermore, Zheng et al.^21^ and Hong et al.^31^ both assessed the effect of GWG after OGTT above the NAM recommendations on the perinatal outcomes. They found that excessive GWG after OGTT was related to increased risks of macrosomia, LGA and cesarean section^21,31^. The finding of these prior reports^21,30,31^ were conformed with our study. We showed that every increased one unit in GWG after OGTT among GDM women was associated with higher risks of macrosomia, LGA and cesarean section, and lower risk of preterm birth. However, Komem et al.^32^ suggested that GWG after OGTT was has no association with the risk of LGA, which was inconsistent with our results. The main possible reason might be due to the fact that Komem et al.^32^ merely classified GDM women to low BMI group (included underweight and normal weight group) and high BMI group (included overweight and obesity group). Besides, it also might be related to different confounders adjustment in different study. We adjusted variables of age, pre-pregnancy BMI, gestational age at birth, GWG before OGTT in this study, and Komem et al.^32^ adjusted variables including maternal age, parity, pre-pregnancy BMI category, treatment modality, OGTT values, chronic hypertension, gestational age at delivery and neonatal gender. In addition, our study suggested that the risk of preterm birth decreased with the increased GWG after OGTT. In contrast, Zheng et al.^21^ reported that GWG after OGTT did not alter the risk of preterm birth, which might be owing to lower prevalence of preterm birth in their studied population than those in this study, with 4.93% versus 8.83%.

Interestingly, our study indicated that positive relationship was found between GWG after OGTT and the SGA probability in underweight and normal weight groups, whereas negative relationship was found in overweight and obesity groups. Saito et al.^33^ found that GDM women with insufficient GWG in overweight/obesity group (BMI ≥ 25 kg/m^2^) was at a higher risk of SGA. They supposed that this might be caused by much lower average GWG in overweight/obesity group and dietary restrictions was conducted among GDM women. However, in our study, average GWG after OGTT in overweight and obesity group was higher than the optimal GWG range proposed by our study. It could be considered as excess GWG. Except for GWG, prior studies^34–36^ considered that pre-pregnancy BMI was a stronger predictor of adverse pregnancy outcomes. Our previous study^16^ found that Chinese GDM women with underweight at a higher risk of SGA. Besides, Apostolopoulou et al.^37^ revealed that higher fat intake and fried foods were both related to higher risk of SGA. In our study, positive association between GWG after OGTT and SGA probability may be related to inadequate micronutrient and unhealthy cooking method.

So far, there is less consensus with respect to the method of exploring the optimal GWG for pregnancies. Several studies^38,39^ modelling the optimal GWG were merely based on fetal growth outcome such as LGA and SGA, however, seldom highlighted maternal adverse outcomes. The optimal GWG for GDM women developed by Cheng et al.^40^ that based on LGA and SGA solely was higher than the GWG proposed by Fan et al.^41^ that was on account of various pregnancy outcomes. This illustrated that fewer adverse outcomes were considered, the greater potential of gaining excessive GWG. Similar with former studies made in China^22^ and Japan^42^, we proposed the optimal GWG after OGTT for GDM women touching on multiple pregnancy adverse outcomes. Such consideration was more feasible for future practice, which optimized pregnancy outcomes through reducing risks of more clinically significant adverse outcomes. Plus, we also performed a quantitative method of RCS to assess the relevance between GWG after OGTT and the predicted probability of adverse outcomes, so that we could more accurately develop the optimal GWG after OGTT for different pre-pregnancy BMI groups.

Importantly, different countries should adopted their own BMI classification standard^22^. Thus, except for considering WHO criteria, we also proposed the optimal GWG after OGTT based on WGOC recommended pre-pregnancy BMI category for Chinese. The two schemes advocated for the similar optimal GWG after OGTT in underweight group and normal weight group. However, for GDM women in overweight group and obesity group, GWG after OGTT based on WGOC criteria had less GWG than those based on WHO criteria. This result was might be supported by prior studies^18,29^ that weight loss after OGTT may be recommended for GDM women with overweight and obesity. The suggestion of weight loss for women with overweight and obesity might be interpreted by following mechanisms. First, overweight and obesity were considered as the independent risk factors for macrosomia, LGA, neonatal asphyxia and cesarean section^43^. Furthermore, maternal obesity seemed to make stronger risk to impact on pregnancy outcomes than GDM, and the combination of obesity and GDM must have the strongest risk of pregnancy adverse outcomes^44^. Accordingly, weight management is crucial for GDM women with overweight and obesity. Besides, our study showed that it is necessary to classified Chinese population based on WOGC recommended pre-pregnancy BMI category, that influenced the contribution of pre-pregnancy BMI groups and the optimal GWG recommendations for GDM women with overweight or obesity.

To our knowledge, this was the first study to explore the optimal GWG after GDM diagnosis for Chinese women with GDM. Strengths of our retrospective study also included that this study had a large sample size, and we also developed the optimal GWG after OGTT for each pre-pregnancy BMI group based on multiple adverse outcomes. Moreover, a key advantage of this study should be considered was that the RCS model was conducted to ensure and improve the fitness of the relationship between GWG after OGTT and the predicted probability of adverse outcomes among GDM women. However, several limitations also need caution. First, as we used limited information exported from medical system, maternal and children’s long-term health outcomes were not be traced. Therefore, the optimal GWG after OGTT proposed by our research might only as guidelines for optimizing short-term pregnancy outcomes. Second, there was no unified approach on how to calculate GWG after OGTT among GDM women retrospectively. Previous study performed by Hong et al.^31^ have used maternal weight at the last prenatal visit prior to delivery minus that at the prenatal visit within 23th–28th gestational weeks of nearest to the OGTT time point. Aiken et al.^30^ directly defined late GWG as wight gain occurred between 28 and 36th gestational weeks. Alternatively, in present study, we calculated GWG after OGTT through weight before delivery at maternity ward minus the average weight at 24th–28th weeks of gestation. Potential bias might be introduced. Thus, a prospective study with large sample size is required in the future for further exploration and validity on the optimal GWG after OGTT among Chinese women with GDM.

In conclusion, our study found that every increased one unit GWG after OGTT among GDM women was associated with higher risks of macrosomia, cesarean section and LGA, and lower risk of preterm birth. According to the WHO and the WGOC recommended pre-pregnancy BMI category, the optimal GWG were proposed: 3.66 to 6.66 kg/3.66 to 6.66 kg in underweight group, 3.07 to 6.50 kg/3.02 to 6.40 kg in normal weight group, 1.06 to 2.73 kg/0 to 1.99 kg in overweight group, and not applicable/− 0.22 to 2.53 kg in obese group, respectively. Moreover, it is necessary to classified Chinese population based on WOGC recommended pre-pregnancy BMI category, that influenced the contribution of pre-pregnancy BMI groups and the optimal GWG recommendations for GDM women with overweight or obesity. The findings of our study provided a reference for clinical medical staff to guide Chinese women with GDM to manage their weight gain from they were diagnosed as GDM so that to promote maternal-infant outcomes.

## Methods

### Study design and participants

In this retrospective study, we extracted medical records of 12,225 GDM women from obstetrical records system from January, 2013 to December, 2018 in a public maternity and children’s hospital in Southeast China. The medical records were measured routinely by trained medical staff during prenatal examinations. The inclusion criteria of participants as follows: (i) GDM pregnant women with single pregnancy; (ii) gestational age at birth greater than 28th weeks. GDM women were excluded from: (i) hyperglycemia was detected before pregnancy but not exclude women have the history of GDM in prior parity; (ii) with chronic diseases such as hypertension, heart disease or kidney disease; (iii) baseline information, GWG data and pregnancy outcomes were incomplete. We collected information including age, ethnics, pre-pregnancy BMI, maternal body weight at diagnosis of GDM, maternal total GWG, maternal GWG before OGTT and after OGTT, gestational age at birth, infant birthweight and perinatal outcomes.

### Diagnosis and management of GDM

All participants attended 75 g OGTT during their 24th–28th gestational weeks. They were diagnosed as GDM based on the criteria proposed by the International Association of Diabetes and Pregnancy Study Group (IADPSG): fasting plasma glucose ≥ 5.1 mmol/L, 1-h plasma glucose ≥ 10.0 mmol/L, or 2-h plasma glucose ≥ 8.5 mmol/L^45^. Once diagnosed, women routinely received standard management including blood glucose monitoring, lifestyle modification, and pharmacological treatment as needed. Besides, the general therapy for GDM^46^ as follows: (i) daily intake of carbohydrates not less than 175 g (daily intake staple food more than 200 g), daily intake of protein not less than 70 g, daily intake of dietary fiber was recommended range 20 g to 30 g; (ii) diversified and low glycemic index (glycemic index less than 55) foods were recommended; (iii) increased in foods rich in iron, folate, calcium, vitamin D, iodine designedly, such as lean meat, poultry, fish, shrimp, dairy products, fresh fruits and vegetables; limited the proportion of foods with high saturated and high fatty acid, such as animal fats, red meat, coconut milk and whole milk products.

### Maternal anthropometrics

Pre-pregnancy BMI was calculated by pre-pregnancy weight (kg) divided by the square of pre-pregnancy height (m^2^), categorized into four groups based on the World Health Organization (WHO) recommendations^23^: underweight (BMI < 18.5 kg/m^2^), normal weight (18.5 kg/m^2^ ≤ BMI < 25.0 kg/m^2^), overweight (25.0 kg/m^2^ ≤ BMI < 30.0 kg/m^2^), and obesity (BMI ≥ 30.0 kg/m^2^). Pregestational anthropometrics were either measured at antenatal examination or self-reported by women during the prenatal examination within the first 12th gestational weeks. Total GWG was refined as women’s weight before delivery minus pre-pregnancy weight. GWG before OGTT was calculated by women’s weight at OGTT minus pre-pregnancy weight. GWG after OGTT was calculated by women’s weight before delivery minus weight at OGTT. Women’s weight before delivery was measured on admission during their expected date of delivery by nurse in obstetrical ward. For weight at OGTT, we took the weight at any week during 24th–28th gestational weeks. If data set of weight during this period greater than one, average weight was calculated and considered as weight at OGTT.

### Pregnancy adverse outcomes

Pregnancy adverse outcomes in this study included macrosomia, preterm birth, cesarean section, gestational hypertension, LGA and SGA. Macrosomia was defined as infant birthweight > 4000 g. Preterm birth was defined as delivery of 28th–37th gestational weeks. Cesarean section represented surgical delivery after 37th gestational weeks with medical indication. Gestational hypertension referred to systolic blood pressure ≥ 140 mmHg and/or diastolic blood pressure ≥ 90 mmHg after 20th gestational week. According to Chinese birth weight curve, LGA and SGA represented infant birthweight below the 10th percentile and above the 90th percentile for gestational age, respectively^47^.

### Statistical analysis

Data preprocessing, data analysis and graphical drawing were performed using R software (Version 3.5.1). Comparisons between pre-pregnancy BMI stratification were performed using One-Way ANOVA for continuous variables and Kruskal–Wallis test or Fisher's exact test for categorical variables, respectively. Three models^48,49^ were used to estimate the relationships between GWG after OGTT and the odds ratio and individual predicted probability of adverse outcomes. (1) Crude model: a crude binary regression model; (2) Logit model: a binary regression model with “logit” link, adjusted for age, pre-pregnancy BMI, gestational age at birth, GWG before OGTT; (3) Complementary log–log model: a binary regression model with “complementary log–log” link, adjusted for variables that same as Model 2. Among adverse outcomes, LGA, SGA were defined in terms of gestational age specific percentile and preterm birth was defined based on gestational age, which thus eliminate the need of adjustment for gestational age in those models^39^. A model with lower Akaike information criterion (AIC) value was regarded as “best-fit model” to develop the optimal GWG after OGTT in different pre-pregnancy BMI groups. Besides, the optimal GWG after OGTT was defined as the range that the lower and upper bounds were the point at which not exceed 1% increase in the predicted probability of adverse outcomes from the lowest point as reported by previous study^48^. Equations to calculate predicted probability of each and total adverse outcome were reported as Eqs. (1) and (2), respectively. Then, to improve the fitness of the association between GWG after OGTT and the predicted probability of adverse outcomes, restricted cubic spline (RCS) model was performed by using R software (Version 3.5.1) packages “rms”, which similar with prior exploration^22^. *P* values < 0.05 was set to indicate statistical significance for two-tailed test.

1 $$\text{Individual predicted probability}= \frac{{\text{exp}}\left({\beta }_{0}+{\beta }_{1}(GWG\, after\, OGTT)+{\beta }_{2}{\overline{x} }_{2}{+\beta }_{3}{\overline{x} }_{3}+{\beta }_{4}{\overline{x} }_{4}+\cdots \right)}{1+{\text{exp}}\left({\beta }_{0}+{\beta }_{1}(GWG\, after\, OGTT)+{\beta }_{2}{\overline{x} }_{2}{+\beta }_{3}{\overline{x} }_{3}+{\beta }_{4}{\overline{x} }_{4}+\cdots \right)}$$

*x* refer to the variables for adjustment in models. GWG, gestational weight gain; OGTT, oral glucose tolerance test

2 $$\text{Predicted probability of adverse outcomes}=\sum _{i=1}^{6}\text{Individual predicted probability of }i$$

*i*_1_: macrosomia; *i*_2_: preterm birth; *i*_3_: cesarean section; *i*_4_: gestational hypertension; *i*_5_: large for gestational age; *i*_6_: small for gestational age.

### Ethics declarations

This study was conducted in accordance with ethical procedures and also approved by the Ethics Committee of Fujian Maternity and Child Health Hospital (No. 2019161). Informed consent was received from each GDM women.

### Supplementary Information

Supplementary Information.

## Data Availability

The datasets generated during and/or analysed during the current study are not publicly available due to they contain information that could compromise the privacy or consent of the research participants but are available from the corresponding author on reasonable request.
