# Supplementary material for: The exploration of optimal gestational weight gain after oral glucose tolerance test for Chinese women with gestational diabetes mellitus
Source: Sci Rep. 2024 Jan 17;14:1466. doi: 10.1038/s41598-024-51879-x (PMC10794465; doi:10.1038/s41598-024-51879-x)
Supplement: Supplementary file 1 — Supplementary Information. [file 41598_2024_51879_MOESM1_ESM.docx]

**Online Supporting Information**

Supplement to: QingXiang Zheng, Yu Zhu, XiuMin Jiang, Ling Huang, JiaNing Li, RuLin Liu. The Exploration of Optimal Gestational Weight Gain after Oral Glucose Tolerance Test for Chinese Women with Gestational Diabetes Mellitus.

**Figure S1 Derivation of the study population**


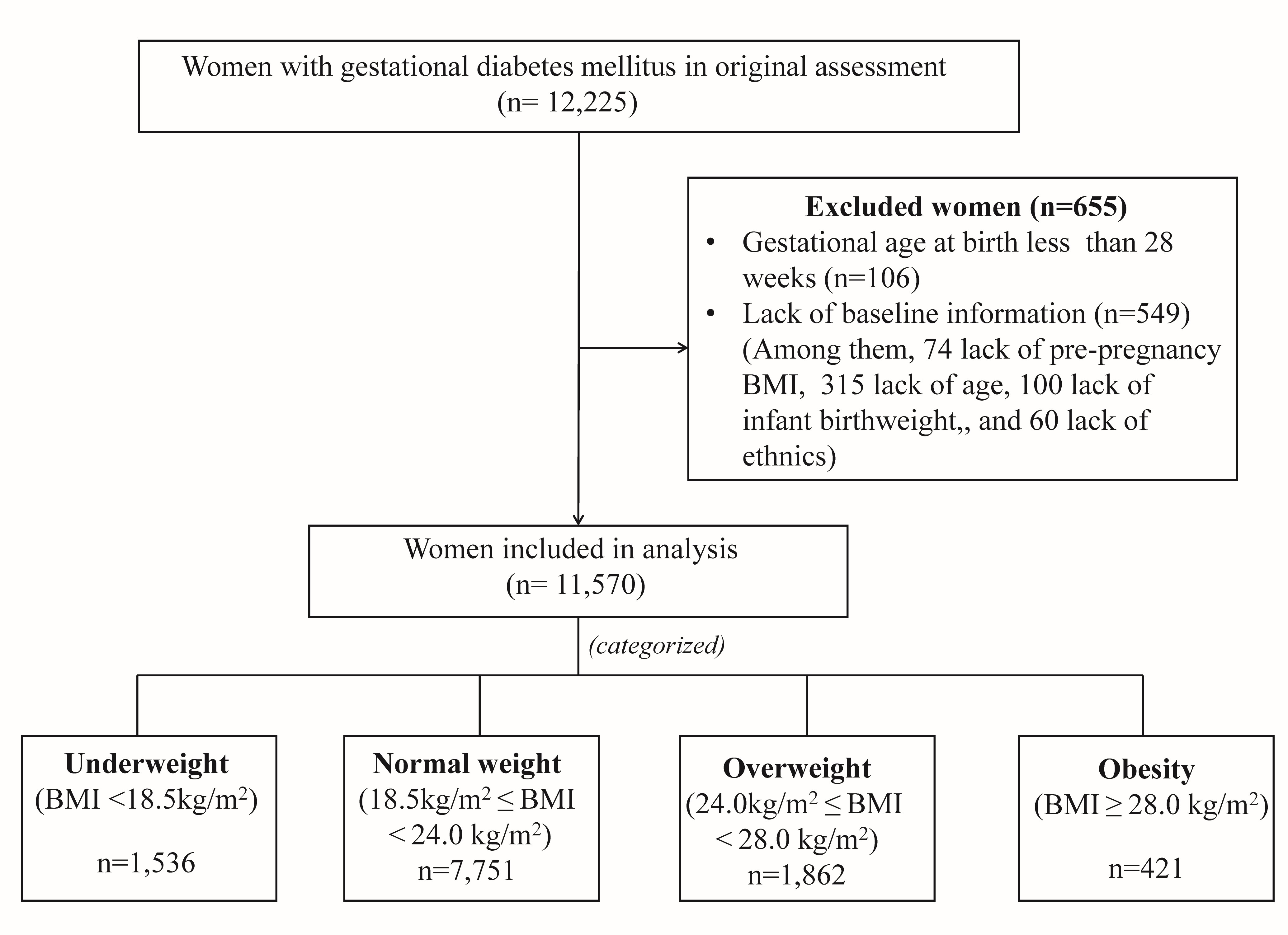


**Table S1 Characteristics of study participants categorized by WGOC BMI criteria**

| **Variables** | **All participants**  (n=11,570) | **Underweight group**  (n=1,536, BMI <18.5kg/m^2^) | **Normal weight group**  (n=7,751, 18.5kg/m^2^ ≤ BMI < 24.0 kg/m^2^) | **Overweight group**  (n=1,862, 24.0kg/m^2^ ≤ BMI < 28.0 kg/m^2^) | **Overweight group**  (n=421, BMI ≥ 28.0 kg/m^2^) | ***P* values** |
| --- | --- | --- | --- | --- | --- | --- |
| Age (year) | 31.00 (28.00, 34.00) | 28.00 (26.00, 32.00) | 31.00 (28.00, 34.00) | 32.00 (29.00, 36.00) | 31.00 (28.00, 35.00) | <0.001 |
| **Ethnics [N (%)]** |  |  |  |  |  |  |
| Han nationality | 11441 (98.89) | 1522 (99.09) | 7658 (98.80) | 1845 (99.09) | 416 (98.81) | 0.642 |
| Minority nationality | 129 (1.11) | 14 (0.91) | 93 (1.20) | 17 (0.91) | 5 (1.19) |  |
| Pre-pregnancy BMI (kg/m^2^) | 21.29 (19.53, 23.25) | 17.72 (17.09, 18.02) | 21.08 (19.92, 22.31) | 25.31 (24.56, 26.30) | 29.49 (28.65, 31.16) | <0.001 |
| Maternal bodyweight at diagnosis of GDM (kg) | 62.00 (56.88, 67.90) | 53.00 (50.05, 55.93) | 61.50 (57.50, 65.55) | 71.00 (66.91, 75.20) | 81.00 (76.97, 86.20) | <0.001 |
| Maternal GWG before OGTT (kg) | 7.50 (5.45, 9.63) | 7.90 (6.20, 9.95) | 7.75 (5.80, 9.90) | 6.20 (4.17, 8.70) | 4.80 (2.30, 7.73) | <0.001 |
| Maternal GWG after OGTT (kg) | 5.00 (3.35, 6.90) | 5.84 (4.20, 7.60) | 5.10 (3.42, 6.90) | 4.30 (2.60, 6.23) | 4.00 (2.20, 5.80) | <0.001 |
| Gestational age at birth (week) | 39.00 (38.00, 40.00) | 39.00 (38.00, 40.00) | 39.00 (38.00, 40.00) | 39.00 (38.00, 40.00) | 39.00 (38.00, 40.00) | 0.348 |
| Infant birthweight (kg) | 3295.00 (3000.00, 3580.00) | 3180.00 (2910.00, 3440.00) | 3295.00 (3000.00, 3575.00) | 3360.00 (3056.00, 3690.00) | 3430.00 (3100.00, 3740.00) | <0.001 |
| **Outcomes [N (%)]** |  |  |  |  |  |  |
| Macrosomia | 638 (5.51) | 32 (2.08) | 384 (4.95) | 171 (9.18) | 51 (12.11) | <0.001 |
| Preterm | 1022 (8.83) | 113 (7.36) | 702 (9.06) | 168 (9.02) | 39 (9.26) | 0.186 |
| Cesarean section | 4169 (36.03) | 356 (23.18) | 2760 (35.61) | 839 (45.06) | 214 (50.83) | <0.001 |
| Gestational hypertension | 187 (1.62) | 10 (0.65) | 107 (1.38) | 51 (2.74) | 19 (4.51) | <0.001 |
| LGA | 1162 (10.04) | 69 (4.49) | 732 (9.44) | 282 (15.15) | 79 (18.76) | <0.001 |
| SGA | 899 (7.77) | 187 (12.17) | 579 (7.47) | 108 (5.80) | 25 (5.94) | <0.001 |

GWG, gestational weight gain; OGTT, oral glucose tolerance test; LGA, large for gestational age; SGA, small for gestational age

**Table S2 The predictive probability of macrosomia, preterm, cesarean section, gestational hypertension, LGA and SGA in association with maternal gestational weight gain after oral glucose tolerance test for underweight, normal weight and overweight GDM women by different model.**

|  | Crude | | | Logit | | | Complementary log-log | | |
| --- | --- | --- | --- | --- | --- | --- | --- | --- | --- |
|  | OR (95%CI) | *P* | AIC | Adjusted  OR (95%CI) | *P* | AIC | Adjusted  OR (95%CI) | *P* | AIC |
| **Underweight** |  |  |  |  |  |  |  |  |  |
| Macrosomia | 1.11 (0.98-1.25) | 0.092 | 312.37 | 1.11 (0.98-1.25) | 0.013 | 314.29 | 1.11 (0.98-1.24) | 0.102 | 314.28 |
| Preterm | 0.24 (0.16-0.35) | <0.001 | 773.18 | 0.81 (0.75-0.87) | <0.001 | 776.86 | 0.84 (0.79-0.89) | <0.001 | 778.84 |
| Cesarean section | 1.06 (1.02-1.11) | 0.008 | 1660.10 | 1.11 (1.06-1.16) | <0.001 | 1626.40 | 1.09 (1.05-1.13) | <0.001 | 1626.10 |
| Gestational hypertension | 1.07 (0.86-1.32) | 0.535 | 124.24 | 1.13 (0.91-1.36) | 0.226 | 125.50 | 1.13 (0.91-1.33) | 0.226 | 125.10 |
| LGA | 1.08 (0.99-1.17) | 0.095 | 564.28 | 1.10 (1.01-1.19) | 0.032 | 554.08 | 1.09 (1.01-1.18) | 0.034 | 554.40 |
| SGA | 1.03 (0.98-1.09) | 0.277 | 1140.60 | 1.02 (0.96-1.08) | 0.612 | 1117.10 | 1.01 (0.96-1.07) | 0.640 | 1116.50 |
| **Normal weight** |  |  |  |  |  |  |  |  |  |
| Macrosomia | 1.11 (1.06-1.15) | <0.001 | 3033.60 | 1.11 (1.06-1.15) | <0.001 | 2860.70 | 1.10 (1.06-1.14) | <0.001 | 2861.00 |
| Preterm | 0.83 (0.81-0.86) | <0.001 | 4556.10 | 0.84 (0.81-0.86) | <0.001 | 4538.80 | 0.86 (0.84-0.88) | <0.001 | 4549.10 |
| Cesarean section | 1.03 (1.01-1.05) | <0.001 | 10088.00 | 1.09 (1.07-1.11) | <0.001 | 9584.60 | 1.07 (1.05-1.09) | <0.001 | 9589.90 |
| Gestational hypertension | 1.10 (1.02-1.17) | 0.008 | 1126.10 | 1.18 (1.10-1.26) | <0.001 | 1089.60 | 1.17 (1.10-1.25) | <0.001 | 1089.80 |
| LGA | 1.03 (1.00-1.06) | 0.025 | 4846.30 | 1.06 (1.03-1.09) | <0.001 | 4650.20 | 1.06 (1.03-1.09) | <0.001 | 4652.00 |
| SGA | 1.05 (1.01-1.08) | 0.004 | 4113.70 | 1.03 (1.00-1.07) | 0.044 | 4050.90 | 1.03 (1.00-1.06) | 0.050 | 4051.20 |
| **Overweight** |  |  |  |  |  |  |  |  |  |
| Macrosomia | 1.15 (1.09-1.21) | <0.001 | 1118.20 | 1.12 (1.06-1.18) | <0.001 | 1058.30 | 1.11 (1.06-1.17) | <0.001 | 1057.90 |
| Preterm | 0.86 (0.81-0.91) | <0.001 | 1102.80 | 0.86 (0.81-0.91) | <0.001 | 1094.80 | 0.87 (0.82-0.92) | <0.001 | 1094.90 |
| Cesarean section | 1.03 (1.00-1.07) | 0.030 | 2562.40 | 1.05 (1.02-1.09) | 0.002 | 2489.80 | 1.04 (1.02-1.07) | <0.001 | 2488.30 |
| Gestational hypertension | 1.03 (0.94-1.13) | 0.529 | 471.15 | 1.07 (0.97-1.17) | 0.176 | 463.53 | 1.07 (0.97-1.16) | 0.171 | 464.52 |
| LGA | 1.08 (1.04-1.13) | <0.001 | 1574.30 | 1.08 (1.04-1.13) | <0.001 | 1521.80 | 1.08 (1.04-1.12) | <0.001 | 1522.50 |
| SGA | 0.99 (0.93-1.06) | 0.772 | 828.54 | 0.98 (0.92-1.04) | 0.513 | 827.36 | 0.98 (0.92-1.04) | 0.509 | 827.34 |
| **Obesity** |  |  |  |  |  |  |  |  |  |
| Macrosomia | 1.14 (1.05-1.25) | 0.003 | 305.66 | 1.12 (1.03-1.23) | 0.011 | 304.21 | 1.11 (1.02-1.19) | 0.009 | 303.99 |
| Preterm | 0.92 (0.86-0.99) | 0.025 | 259.08 | 0.92 (0.86-0.99) | 0.025 | 263.97 | 0.94 (0.90-1.00) | 0.032 | 264.54 |
| Cesarean section | 1.02 (0.97-1.07) | 0.458 | 586.95 | 1.02 (0.97-1.08) | 0.433 | 567.71 | 1.02 (0.98-1.06) | 0.395 | 567.04 |
| Gestational hypertension | 1.06 (0.94-1.21) | 0.383 | 158.08 | 1.13 (0.98-1.30) | 0.081 | 146.06 | 1.11 (0.97-1.25) | 0.101 | 146.56 |
| LGA | 1.12 (1.04-1.21) | 0.004 | 401.80 | 1.11 (1.03-1.20) | 0.008 | 394.50 | 1.09 (1.02-1.16) | 0.009 | 394.53 |
| SGA | 0.94 (0.87-1.03) | 0.157 | 191.99 | 0.95 (0.87-1.04) | 0.194 | 192.09 | 0.95 (0.90-1.04) | 0.213 | 192.24 |

Data are represented as OR (95% CI). ORs were calculated by binary regression model using crude, “logit” and “complementary log-log” links, and were adjusted for age, pre-pregnancy BMI, gestational age at birth, GWG before OGTT. Particularly, gestational age at birth was not adjusted in LGA, SGA and preterm models.

OR, odds ratio; CI: confidence interval; AIC, Akaike information criterion; GWG, gestational weight gain; OGTT, oral glucose tolerance test; LGA, large for gestational age; SGA, small for gestational age. Underweight, normal weight, overweight and obesity were defined as: pre-pregnancy BMI <18.5kg/m^2^, 18.5kg/m^2^≤ BMI < 24.0 kg/m^2^, 24.0kg/m^2^ ≤ BMI < 28.0 kg/m^2^ and BMI ≥ 28.0 kg/m^2^, respectively.


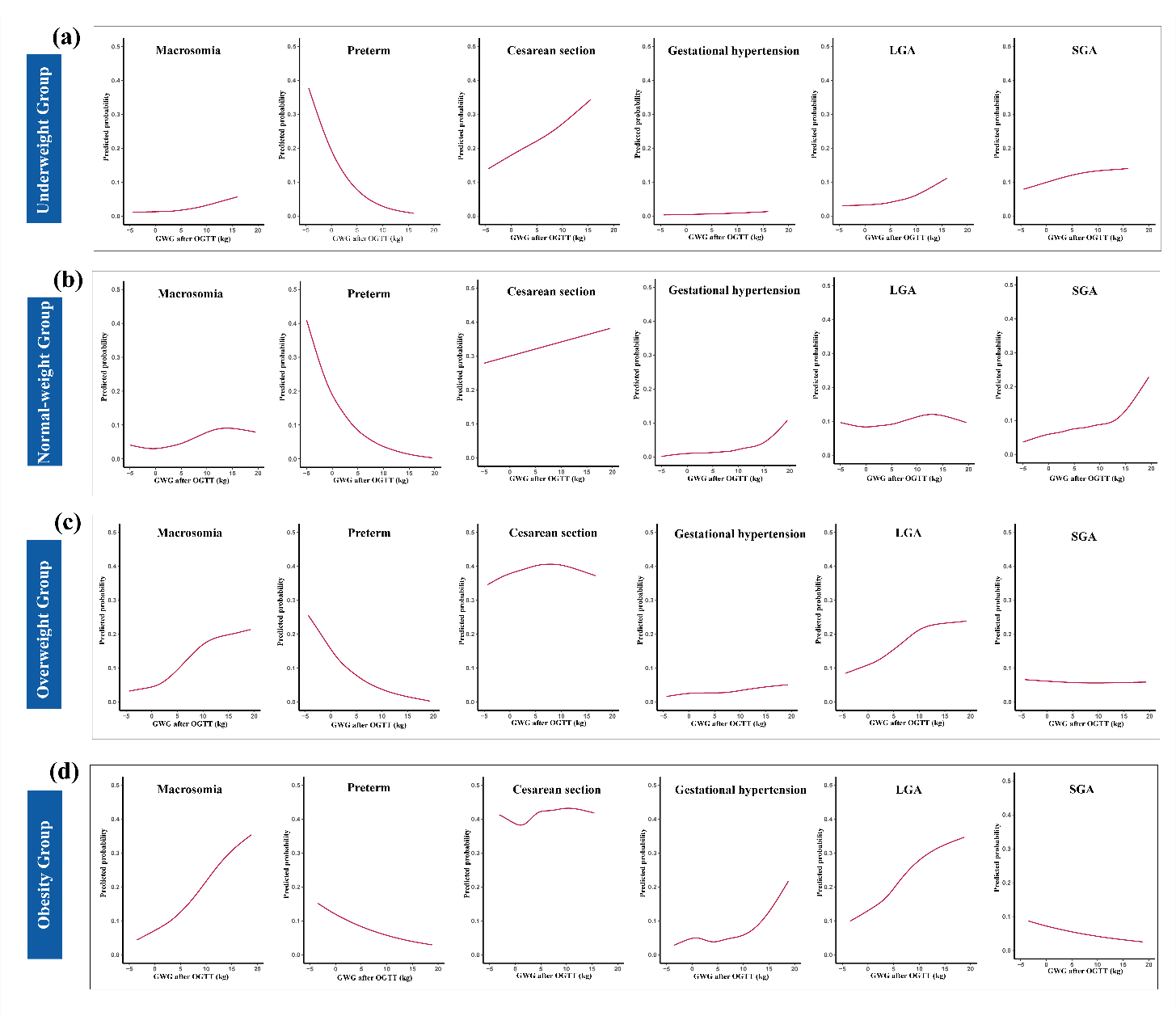
**Figure S2 Association between gestational weight gain after oral glucose tolerance test and the predicted probability of adverse outcomes in different pre-pregnancy BMI category.**

Binary regression model with low Akaike information criterion value was used to calculate predicted probability of adverse outcomes including macrosomia, preterm birth, cesarean section, gestational hypertension, LGA and SGA.

GWG, gestational weight gain; OGTT, oral glucose tolerance test; LGA, large for gestational age; SGA, small for gestational age

(a)Underweight group; (b) Normal weight group; (c) Overweight group; (d) Obesity group
